# Occupational radiation exposure in femoral artery approach is higher than radial artery approach during coronary angiography or percutaneous coronary intervention

**DOI:** 10.1038/s41598-020-62794-2

**Published:** 2020-04-28

**Authors:** Jung-Su Kim, Bong-Ki Lee, Dong-Ryeol Ryu, Kwangjin Chun, Ho-Seok Kwon, So-Ra Nam, Doo-il Kim, Sung-Yun Lee, Jin-Ok Jeong, Jang-Whan Bae, Jong-Seon Park, Youngkeun Ahn, Je-Keon Chae, Myeong-Ho Yoon, Seung-Hwan Lee, Jeonghan Yoon, Hyeon-Cheol Gwon, Donghoon Choi, Soon-Mu Kwon, Young-Hoon Roh, Byung-Ryul Cho

**Affiliations:** 1 Department of Radiologic technology, Daegu Health College, Daegu, Korea; 2 Division of Cardiology, Department of Internal Medicine, Kangwon National University Hospital, Kangwon National University, School of Medicine, Chuncheon, Korea; 3 Department of Health and Safety Convergence Science, College of Health Science, Korea University, Seoul, Korea; 4 Division of Cardiology, Department of Internal Medicine, Inje University Haeundae Paik Hospital, Inje University, College of Medicine, Busan, Korea; 5 Division of Cardiology, Department of Internal Medicine, Inje University Ilsan-Paik Hospital, Inje University, College of Medicine, Goyang, Korea; 6 Division of Cardiology, Department of Internal Medicine, Chungnam National University, School of Medicine, Daejeon, Korea; 7 Division of Cardiology, Department of Internal Medicine, College of Medicine, Chungbuk National University, Cheongju, Korea; 8 Division of Cardiology, Department of Internal Medicine, Yeungnam University Hospital, Yeungnam University, School of Medicine, Daegu, Korea; 9 Division of Cardiology, Department of Internal Medicine, Chonnam National University Hospital, Chonnam National University, School of Medicine, Chonnam, Korea; 10 Division of Cardiology, Department of Internal Medicine, Chonbuk National University Hospita, Chonbuk National University, School of Medicine, Chonbuk, Korea; 11 Division of Cardiology, Department of Internal Medicine, Ajou University Hospital, Ajou University, School of Medicine, Kyeonggi, Korea; 12 Division of Cardiology, Department of Internal Medicine, Yonsei University Wonju, College of Medicine, Wonju, Korea; 13 Division of Cardiology, Department of Medicine, Samsung Medical Center, Sungkyunkwan University, School of Medicine, Seoul, Korea; 14 Division of Cardiology, Severance Cardiovascular Hospital, Yonsei University College of Medicine, Seoul, Korea

**Keywords:** Interventional cardiology, Radiography

## Abstract

Medical radiation exposure is a significant concern for interventional cardiologists (IC). This study was aimed at estimating the radiation exposure of IC operators and assistants in real clinical practice. The radiation exposure of the operator and assistant was evaluated by conducting two types of procedures via coronary angiography (CAG) and percutaneous coronary intervention (PCI) on 1090 patients in 11-cardiovascular centers in Korea. Radiation exposure was measured using an electronic personal dosimeter (EPD). EPD were attached at 3 points on each participant: on the apron on the left anterior chest (A1), under the apron on the sternum (A2), and on the thyroid shield (T). Average radiation exposure (ARE) of operators at A1, A2, and T was 19.219 uSv, 4.398 uSv, and 16.949 uSv during CAG and 68.618 uSv, 15.213 uSv, and 51.197 uSv during PCI, respectively. ARE of assistants at A1, A2, and T was 4.941 uSv, 0.860 uSv, and 5.232 uSv during CAG and 20.517 uSv, 4.455 uSv, and 16.109 uSv during PCI, respectively. AED of operator was 3.4 times greater during PCI than during CAG.

## Introduction

The use of ionizing radiation in invasive cardiology procedures such as coronary angiography (CAG) and percutaneous coronary intervention (PCI)^1^ is customary; however, during the last 10 years, issues related to radiation hazards and injury have been raised, which increases the need to include long-term cancer risk due to ionizing radiation in the risk-benefit assessment of diagnostic or therapeutic procedures^2^. Medical radiation exposure is a significant concern for interventional cardiologists because the workload and complexity of procedures have increased over the past few years without a corresponding increase in the number of interventional cardiologists^3^, who represent the most important group of medical specialists involved in medical radiation practices. According to a report published by the International Commission on Radiological Protection (ICRP) that discussed the importance of radiation protection of patients and medical staff in the interventional cardiovascular field^4^, interventional procedures may increase the risk of skin injury or cancer to both the patient as well as the staff. Several aspects of medical radiation safety in the practice of interventional cardiology have been addressed by the American College of Cardiology in a consensus document^5^. According to the UNSCEAR 2000 report of the United Nations, fluoroscopic procedures are the largest source of occupational radiation exposure in medicine by far^6^. The purpose of our study was to monitor and estimate the occupational radiation exposure of interventional cardiology operators and assistants during coronary angiography (CAG) and percutaneous coronary intervention (PCI) procedures in real clinical practice at the Korean Cardiovascular Center.

## Results

This study was performed on 682 male and 408 female patients, between 28 and 102 years of age, with an average age of 66.09. The weight of the patients ranged from 24.19 kg to 103 kg, with an average of 64.42 kg, and their height was between 126.00 cm and 188.00 cm, the average being 161.50 cm. The subjects were divided into two groups: the first group received the CAG procedure and the second group received the PCI procedure.

**Figure 1:**
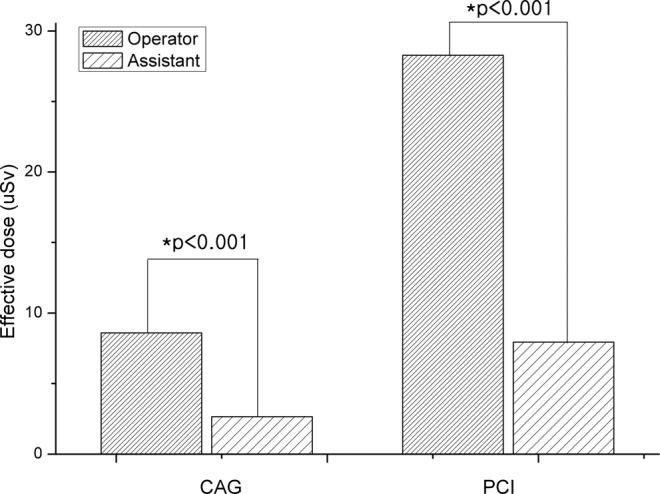
Effective dose of CAG and PCI procedures for cardiology operator and assistants.

**Figure 2:**
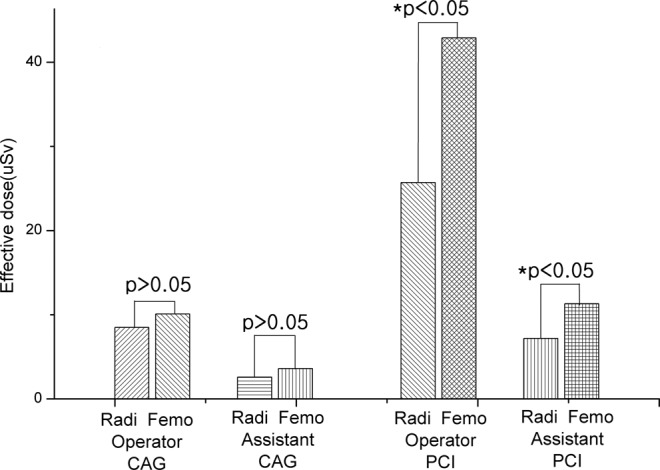
Comparison of average effective dose in radial and femoral artery approaches in CAG and PCI procedures, between cardiology operator and assistants.

The CAG procedure was carried out on 801 patients and the PCI procedure was carried out on 289 patients. In the CAG procedure, the average exposure doses at A1, A2, and T to the operator were found to be 19.219 µSv, 4.398 µSv, and 16.949 µSv, respectively; the average exposure doses at A1, A2, and T to the assistant were 4.941 µSv, 0.860 µSv, and 5.232 µSv, respectively (Table 1). The average ED in CAG procedures was 8.584 µSv and 2.638 µSv to operator and assistant, respectively (Fig. 1). The average ED to operator and assistant in transfemoral artery approach procedures was 10.101 µSv and 3.592 µSv, respectively, showing that the average ED to operator is 2.5 times higher than that to assistant. The average ED to operator and assistant in transradial artery approach procedures was 8.490 µSv and 2.579 µSv, respectively, indicating that it is 3.3 times higher for operator than for assistant(Table 2, Fig. 2). The average ED to operator in the transfemoral artery approach procedures is 18.98% higher than that in the transradial artery approach procedures. In the case of the average ED to assistant, it is 39.29% higher in the transfemoral artery approach procedures than in the transradial artery approach procedures. Patient average cumulative fluoroscopy time during the CAG procedure was 236.27 sec and 311.89 sec in the transradial artery approach and the transfemoral artery approach, respectively (Table 3). Patient average radiation exposure doses for the transfemoral artery approach and the transradial artery approach was summarized in Table 3.

**Table 1:** Average effective dose of interventional cardiology operator & assistants in femoral & radial vessel approach procedures.

		CAG		PCI	
		Operator ED (µSv)	Assistant ED (µSv)	Operator ED (µSv)	Assistant ED (µSv)
Femoral artery approach	Average	10.101	3.592	42.888	11.298
	Standard deviation	10.892	4.852	50.192	13.059
	Minimum	0.100	0.050	0.750	0.300
	Maximum	39.560	24.370	249.870	54.660
Radial artery approach	Average	8.490	2.579	25.694	7.179
	Standard deviation	11.771	6.317	27.858	8.295
	Minimum	0.050	0.050	0.200	0.050
	Maximum	167.980	140.420	160.640	62.470
Femoral & radial artery approach	Average			69.460	32.160
	Standard deviation			70.097	27.476
	Minimum			10.620	7.650
	Maximum			163.790	71.050

CAG: coronary angiography, PCI: percutaneous coronary intervention, ED: effective dose.

**Table 2:** Measurement values (µSv) of exposure dose for each dosimeter.

Procedure		Operator A1	Operator A2	Operator T	Assistant A1	Assistant A2	Assistant T
CAG	Average	19.219	4.398	16.949	4.941	0.860	5.232
	Standard deviation	30.808	12.657	23.235	8.208	2.028	12.443
	Minimum	0.060	0.010	0.010	0.010	0.010	0.040
	Maximum	463.830	256.470	331.050	133.590	29.310	280.000
PCI	Average	68.618	15.213	57.193	20.571	4.455	16.109
	Standard deviation	79.385	30.860	67.029	35.583	9.932	19.905
	Minimum	0.400	0.100	0.380	0.130	0.030	0.100
	Maximum	497.6400	319.800	491.470	372.890	79.980	141.000

CAG: coronary angiography, PCI: percutaneous coronary intervention.

**Table 3:** Average patient radiation dose in transfemoral & transradial artery approach procedures.

		CAG				PCI			
		Cum Fluoro time (sec)	Cum Fluoro KAP (Gy·cm^2^)	Cum Exposure KAP (Gy·cm^2^)	Total KAP (Gy·cm^2^)	Cum Fluoro time (sec)	Cum Fluoro KAP (Gy·cm^2^)	Cum Exposure KAP (Gy·cm^2^)	Total KAP (Gy·cm^2^)
Transfemoral artery approach	Average	311.89	20.73	25.79	46.95	1348.34	103.17	60.97	154.65
	Standard deviation	265.42	15.64	14.84	21.90	1188.16	118.48	48.50	158.47
	Minimum	74.00	3.04	5.52	10.56	65.00	3.47	8.56	13.98
	Maximum	1585.00	71.03	99.21	107.19	5479.00	539.46	232.53	711.89
Transradial artery approach	Average	236.27	17.41	22.95	39.99	990.69	78.80	47.93	122.74
	Standard deviation	247.83	23.83	14.52	32.53	703.61	99.03	31.05	102.94
	Minimum	32.00	0.01	1.68	0.30	117.00	0.04	0.02	0.06
	Maximum	2776.00	368.01	140.67	508.68	4410.00	1117.87	188.10	796.88
Transfemoral & transradial artery approach	Average					3096.25	132.25	135.77	204.37
	Standard deviation					2266.15	73.30	87.22	173.86
	Minimum					984.00	78.49	35.05	13.44
	Maximum					5465.00	215.74	186.69	401.29

CAG: coronary angiography, PCI: percutaneous coronary intervention, Cum Fluoro time: cumulated fluoroscopy time, Cum Fluoro KAP: cumulated fluoroscopy kerma area product, Cum Exposure KAP: cumulated exposure kerma area product, Total KAP: total kerma area product.

On the PCI procedure, average exposure doses at A1, A2, and T to operator were 68.618 µSv, 15.213 µSv, and 57.193 µSv, respectively, while those to assistant were 20.571 µSv, 4.455 µSv, and 16.109 µSv, respectively (Table 1). The average ED in PCI procedures was 28.977 µSv and 8.166 µSv to operator and assistant, respectively (Fig. 1). The average ED to operator and assistant in transfemoral artery approach procedures was 42.888 µSv and 11.298 µSv, respectively, i.e., it is 3.8 times higher for an operator than for an assistant. The average ED to operator and assistant in the transradial artery approach procedures was 25.694 µSv and 7.179 µSv respectively; hence, for an operator, it is 3.6 times higher than for an assistant. In the case of the transradial artery and the transfemoral artery approach procedures taken together, the average ED to operator is 2.2 times higher than that to assistant (Table 2, Fig. 2). In the PCI procedure, the patient average cumulative fluoroscopy time for the transfemoral artery approach and the transradial artery approach was 1348.34 sec and 990.69 sec respectively (Table 3).

**Figure 3:**
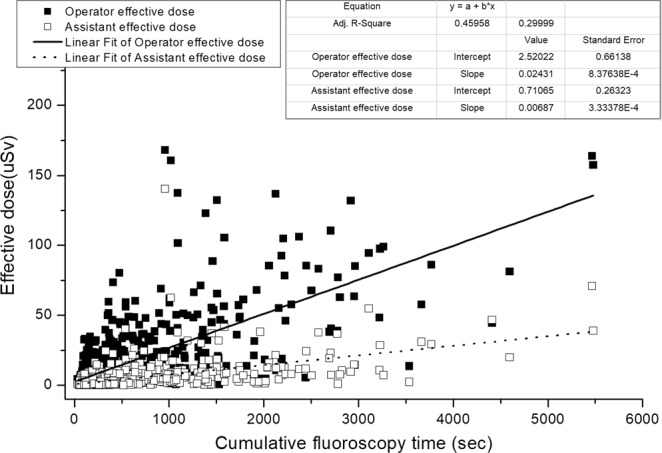
The interaction equation of cumulative fluoroscopy time of a patient with average ED to operator and assistant.

By correlation analysis, the correlation coefficient between the average ED and approach vessel was found to be −0.131 (p < 0.01) and −0.077 (p < 0.5) to operator and assistant, respectively. (Fig. 3). Both correlation coefficients show low significant connection between the average ED to operator and assistant and the approach vessel.

**Figure 4:**
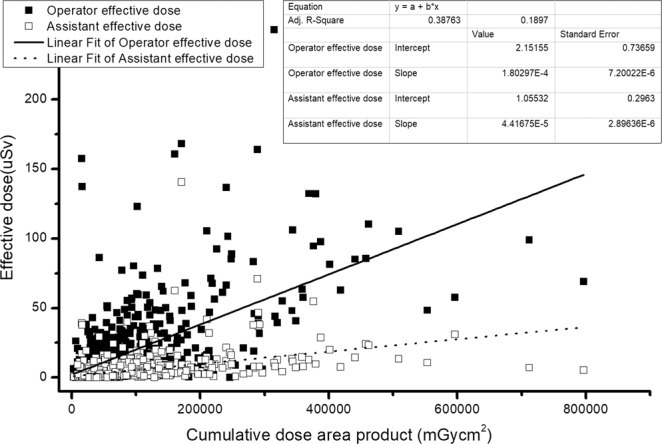
The interaction equation of cumulative dose area product of a patient with average ED to operator and assistant.

The correlation coefficients between the cumulative fluoroscopy time and the product of cumulative dose per patient and average ED to operator and assistant are 0.678 (0.01 < p), 0.548 (0.01 < p), 0.629 (0.01 < p), and 0.453 (0.01 < p), respectively (Fig. 4). All the correlation coefficients show a strong significant correlation.

## Discussion

There is great concern about the potential effects of occupational radiation exposure on interventional cardiology staff and assistants performing CAG and PCI procedures, as they are exposed to high radiation levels. Therefore, it was necessary to estimate the ED to operator and assistant during these procedures. According to the results obtained, the average ED to operator in CAG and PCI procedures was 8.584 µSv and 28.977 µSv, respectively. If an operator undergoes four procedures per week, the annual estimated ED would be equivalent to approximately 8.204 mSv for CAG and 27.817 mSv for PCI. By the same estimation, the annual ED to an assistant would be 2.532 mSv for CAG and 7.839 mSv for PCI. According to the ICRP publication 103, the annual occupational radiation exposure limit should be under 50 mSv in a year with an average of 100 mSv in 5 years^7,8^. The results of this study show that the average ED to an operator in PCI may exceed 100 mSv in 5 years. In a situation of planned exposure, the revised equivalent dose limit for the lens of the eye is 20 mSv per year which is the average over 5 consecutive years (i.e., 100 mSv in 5 years), and 50 mSv in any single year^9,10^. In this study, the T exposure dose is equal to the exposure dose of the unprotected eye and thyroid. The eye and thyroid are sensitive organs for radiation exposure; hence, the annual equivalent dose for these was assumed to be 16.290 mSv per year. This value is approximately equal to the annual dose limitation of the eye of 20 mSv per year.

Radiation doses measured in this study are comparable to those reported in previous studies.

The results of this study show that the average ED to operator and assistant are higher than those reported in the previous studies of 2010^11–14^. On the CAG, ED of operator in this study was similar to the 2014 study of Georgios Christopoulos *et al*. but over half^15,16^ when compared to the Eltigani Abdelaal *et al*. study and Helmut W. Lange *et al*. study. Additionally, on the PCI, ED of operator in this study was 141% higher than in the Georgios Christopoulos *et al*. study^17^.

This study shows that operator exposure in transfemoral artery approach procedures was higher than that of transradial artery approach procedures. This matches the tentative results of the Lange *et al*. study^12,18^ and the Michael *et al*. study^19^.

This result shows that other factors such as distance from radial source and fluoroscopy time have greater impact on operator and assistant exposure dose than the insertion sites.

Operator exposure can be reduced by increasing the distance from the x-ray source (inverse-square law) and by reducing operator and patient exposure time. In 2006, Marque, N *et al*. studied the effect of the extension tube on operator radiation exposure during coronary procedures performed through the radial artery approach^20^. As a result, a non-significant trend towards lower left-arm operator exposure was noted in the extension catheter group (28.7 ± 31.0 μSv vs 38.4 ± 44.2 μSv, *p* = 0.0739). No significant difference was noted in relation to the type of procedure. In this study of real clinical patient observations, fluoroscopy times and keram area products in the transfemoral artery approach procedure were longer and higher than that of transradial artery approach procedures; the results followed an increase in patient and operator exposure dose.

Lange HW *et al*.’ study and Michael TT study showed that radiation exposure in the transradial artery approach was higher than that in the transfemoral artery approach^18,19^. Michael TT *et al*. reported that transradial artery approach diagnostic CAG was associated with operator radiation exposure compared with transfemoral angiography in patients who had previously undergone CABG surgery. In this study, transradial artery approaches had longer fluoroscopy times than transfemoral artery approaches(8.5 ± 4.7 min vs. 12.7 ± 6.6 min, p < 0.01)^19^. Also, in the study of Lange HW *et al*. although it is not the main aim of this study, fluoroscopy time with the transradial artery approach was longer than that of the transfemoral artery approach (2.7 ± 1.4 min vs. versus 2.1 ± 1.1 min, p < 0.001) and operator radiation dose with standard protection was 20.9 ± 13.8 μSv in the transradial artery approach group and 15.3 ± 10.4 μSv in the femoral artery approach group (p < 0.001).[20] Consequently, fluoroscopic time may be the one of several potent factors determining radiation exposure. Under real clinical conditions, the transfemoral artery approach was done in patients with complex and unfavorable conditions. In 2013, Wimmer NJ *et al*. analyzed the real world data from patients who underwent PCI without intra-aortic balloon pump or other mechanical support at 5 institutions in Massachusetts using either transfemoral or transradial arterial access, In this study, the transfemoral approach is used more frequently in patients with prior MI, prior stroke, prior CPI, prior CABG, peripheral artery disease, dialysis, cardiogenic shock, and emergency situation^21^. These unfavorable conditions usually result in longer procedure times. Therefore, the results of our study explain why the transfemoral artery approach had higher operator radiation doses than the transradial artery approach.

In addition, a significant link is found relating the cumulative dose area product and cumulative fluoroscopy time with the occupational radiation dose measured by the electronic personal dosimeters (EPD) and ED. Occupational radiation dose measurement is an important part of reducing radiation exposure of operator and assistant. It is evident from the results that the operator can lower their own level of risk if they are aware of the need for radiation exposure reduction for their patients. ED to the operator and assistant is mainly the scattered radiation from patients. Thus, if patients receive less radiation, then the operator and assistant will also be exposed to less scattered radiation.

The limitation of this study is that dividing the RT and LT sides was not considered in transradial artery approach procedures. In the Javier Fernandez-Portales *et al*. study, it was shown that anatomic differences of RT and LT sides of the transradial artery could influence the procedure complex, and also affect the success rate and duration of the procedures. From that point of view, dividing the RT and LT sides is not necessary in transradial artery approach procedures because the average cumulative fluoroscopy time was used in this study.

Our next plan is for the continuous monitoring of occupational radiation exposure in interventional cardiology staff and assistants and in-patient radiation exposure doses during CAG and PCI.

## Conclusions

The average ED of operator and assistant during PCI was about 3.4 and 3.1 times greater than during CAG. For the CAG procedures, average ED of operator and assistant between transradial approach and transfemoral approach had similar values. For the PCI procedures, average ED of operator and assistant via transfemoral approach was about 2 times higher than via transradial approach. In medical radiation practice, reducing radiation exposure for both patients and operators is a universal goal. As a result of this study, the occupational radiation dose to the interventional cardiology operator and assistant was found to have various values. Therefore, interventional cardiology operator and assistant should use the appropriate protection devices for CAG and PCI procedures, be aware of radiation effects, and make efforts to reduce the radiation exposure dose for both the operator and assistant.

## Methods

The subjects in this study were 1090 patients on whom coronary angiography (CAG) and percutaneous coronary intervention (PCI) procedures were carried out between August 2016 and October 2017 in 11 cardiovascular centers of university hospitals in Korea.

Occupational radiation exposure dose to operator and assistant was determined using the electronic personal dosimeters (SPD-9100, SFT technology, Korea): the first one was worn on the trunk of the body under the apron (A2), the second outside the apron (A1) at the level of the sternum on the chest, and a third one was worn on the outside of the thyroid protector (T). Each dosimeter was corrected by the Korea Research Institute of Standards and Science and the dosimeter correction factor, k, was 1.00–1.01, with an uncertainty of 7.4%. The dosimeter accuracy was +10% to −10%. The dosimeter under the apron provided an estimated dose, A2, to the organs of the shielded region while that worn outside the thyroid protector, T, provided the estimated dose to the organs of the head and neck, including the thyroid and eye lenses. The effective dose was calculated by substituting the values of A2 and T in Eq. 1. The NCRP report 122 published specific recommendations for calculating the effective dose (ED) when protective aprons were worn during diagnostic and interventional medical procedures^22^.

1 $${\rm{Effective}}\,{\rm{dose}}=0{\rm{.025T}}+0{\rm{.5A2}}$$

The frequency and bivariate correlation analyses were performed using SPSS Version 22 software. (IBM Corporation, USA) This study complied with the Portability and Accountability Act and was approved by the respective institutional review boards of each hospital. This study was approved by institutional review boards of Kangwon national university and approval number of this study is KNUIRB-2015-11-004-003. All participants provided written informed consent.
